# Conformity of Three Pre-Contoured Clavicular Plates Compared Using Personalized 3D-Printed Models of Clavicles from Patients

**DOI:** 10.3390/life14070888

**Published:** 2024-07-18

**Authors:** Hyun Seok Song, Yongwon Joh, Hyungsuk Kim

**Affiliations:** Department of Orthopedic Surgery, Eunpyeong St. Mary’s Hospital, College of Medicine, The Catholic University of Korea, Seoul 03312, Republic of Korea; hssongmd@catholic.ac.kr (H.S.S.); dngdae2@naver.com (Y.J.)

**Keywords:** shoulder, anatomical plate, pre-contoured plate, 3D printing, fracture

## Abstract

The human clavicle’s unique S-shaped, three-dimensional structure complicates fracture management. This study evaluated the anatomical conformity of pre-contoured anatomical plates using 3D-printed clavicle models. CT scans from 30 patients (15 males and 15 females) were used to create these models. Three brands of distal clavicle plate systems (Acumed, Synthes, and Arthrex) were tested for fit. Measurements included the distance from the distal end of the clavicle to the plate’s lateral end, the gap between the clavicle and the plate, and the overhang distance. Results showed significant differences in clavicle length between sexes, with men having a mean length of 156.1 ± 7.6 mm and women 138.4 ± 4.3 mm, both with normal distribution (*p* > 0.05). The mean lateral distance was 7.9 ± 1.7 mm, and the mean medial gap was 3.6 ± 3.0 mm, showing no significant differences between products or sexes. The mean overhang distance was 5.8 ± 4.6 mm, with larger values in women for the Acumed (*p* = 0.037) and Arthrex (*p* = 0.000) plates. Overall, pre-contoured plates exhibited notable discrepancies, especially in shorter clavicles.

## 1. Introduction

Clavicle fractures account for 2% to 5% of all adult fractures and are particularly common within shoulder girdle fractures, which comprise 35% to 44% of such injuries [1,2,3,4]. Traditionally, clavicle fractures were treated nonoperatively with figure-of-eight bandages, even in cases with substantial displacement. This approach was supported by early studies indicating nonunion rates of less than 1% with conservative treatment and relatively high nonunion rates following operative treatment [5,6,7]. However, recent studies report several complications of conservative treatment such as shortening, malunion, limitation of motion of the shoulder joint, and poor patient satisfaction due to long period of motion restriction. These studies suggest operative treatment, which provides firm internal fixation, resulting in an early recovery of the range of motion.

The human clavicle is highly variable within any given population. It is a double-curved, S-shaped, three-dimensional structure with complex morphology. Variations exist not only in its length and diameter but also in its cross-sectional shape and the degree of bowing. Differences are observed between males and females and even between the left and right clavicle in the same individual. This indicates significant personal deviation in clavicle anatomy [8,9,10,11].

Surgical treatments for clavicle fractures include interfragmentary screw fixation, cerclage wiring, intramedullary fixation, and plate fixation [12,13,14,15]. Plate fixation provides rigid fixation, allowing for early mobilization [16,17]. It is particularly preferred in displaced comminuted fractures where soft tissues impede fracture reduction. Plate fixation also offers firm stabilization through cortical bone compression and resistance to torque. One meta-analysis study reported that plate fixation reduced the nonunion rate from 15.1% to 2.2% [18]. However, due to the unique anatomic diversity, clavicle fractures are still challenging injuries to shoulder surgeons.

Pre-contoured anatomical plates were introduced to address these challenges by fitting the natural shape of the clavicle. The original purposes of the pre-contoured plates are to shorten operation time by eliminating the step of contouring the plate at the time of surgery, reducing the possibility of malreduction resulting in malunion afterwards, and preventing skin protrusion by improper fitting of the plate. However, surgeons often find that these plates do not perfectly conform to the clavicle’s unique anatomy, necessitating additional manipulation and bending during surgery.

The application of 3D printing technology in medicine has significantly advanced over recent decades [19,20,21,22]. In orthopedics, 3D printing is used for creating anatomical models primarily for surgical planning, as well as for producing surgical guides and custom implants. Surgical guides and custom implants created with 3D printing technology have become widely used and continue to develop rapidly [8,23,24,25]. These custom implants and guides offer advantages such as reduced surgical time, improved clinical outcomes, and decreased radiation exposure [26].

The aim of this study is to evaluate the conformity of pre-contoured anatomical plates to the human clavicle and to analyze the variations in clavicle shape and length between individuals using 3D-printed clavicle models. Our hypothesis was that, due to the significant anatomic variance in human clavicles, the three widely used commercially available pre-contoured anatomical plates would show a range of conformity with the actual human clavicle.

## 2. Materials and Methods

### 2.1. Patient Enrollment

In this study, 15 male and 15 female patients who had previously undergone three-dimensional shoulder computed tomography (CT) for reasons other than clavicle fractures were randomly selected and matched by sex and age from the existing records at a single hospital. Patients without a full-length image of the clavicular were excluded.

The study protocol received approval from our Institutional Review Board (IRB) (approval number: PC20RISI0021). Given the retrospective design of this study, the IRB waived the need for informed consent.

### 2.2. 3D Modeling

**Figure 1:**
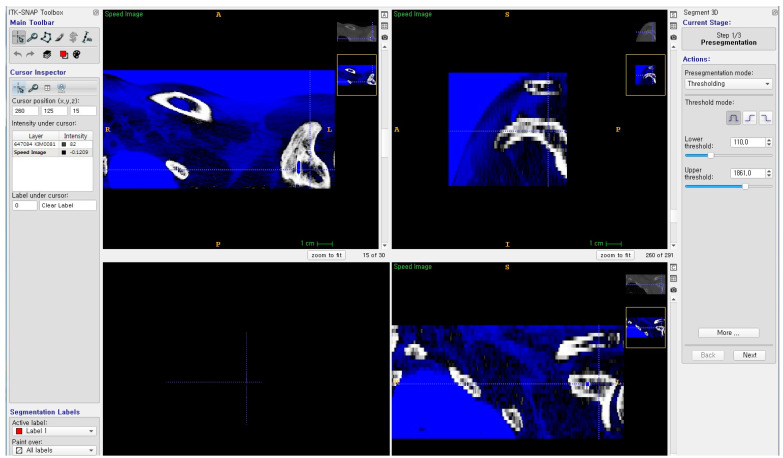
ITK snap (ver 3.4.0) was used for reconstructing CT axial images to a 3D structure image.

CT axial image data were obtained from the PACS (picture archiving and communication system) in DICOM (digital imaging and communications in medicine) format. Free, open-source software, ITK snap (http://www.itksnap.org (accessed on 16 June 2021), ver 3.4.0), was used to reconstruct the CT axial images into a 3D model (Figure 1). Using the program, native 3D anatomical structures were parsed from CT data into STL file format for CAD (computer-aided design) modeling. The semi-automatic segmentation with active contour method was employed in this software. For the pre-segmentation mode, thresholding was utilized with a lower threshold of 110 and the upper threshold set to maximum. The region competition force parameter was set to 0.75. These values were determined based on our experience in order to produce the most efficient and accurate 3D model. Additional manual segmentation was necessary for thin cortical bone areas that were too fine to be accurately identified with the semi-automatic method.

**Figure 2:**
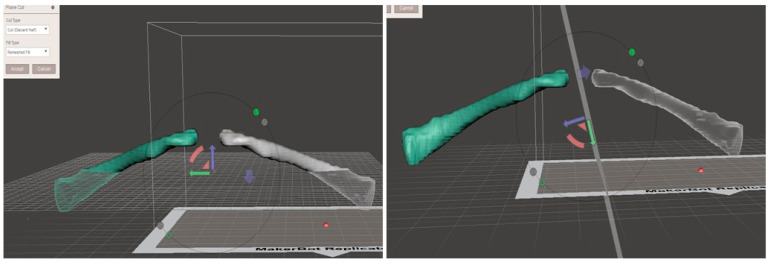
Meshmixer (ver 3.0) was used to invert the left clavicle to the right clavicle.

**Figure 3:**
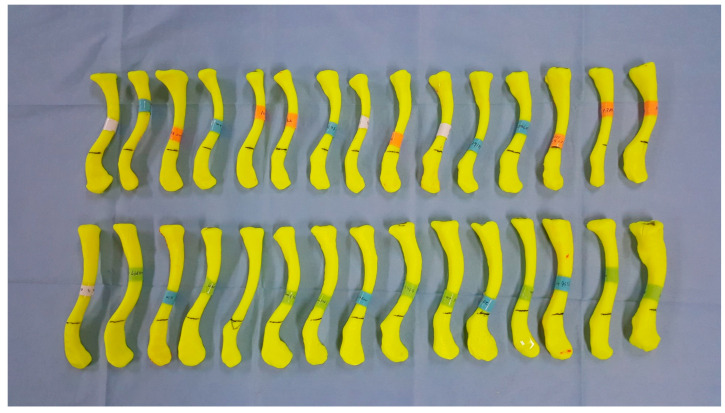
3D clavicle models showing variability of size and curvature.

For left clavicle images, they were converted to right clavicle images using the mirroring function in the free, open-source software Meshmixer (http://www.meshmixer.com, ver 3.0) (Figure 2). All subjects were standardized as right clavicles to avoid measurement bias by using the same right-side plate. The clavicle models were then constructed via an FDM (fused deposition modeling) 3D printer (da Vinci 2.0A; XYZ printing, Taiwan) using ABS (acrylonitrile butadiene styrene)-based filament (0.1 mm layer thickness, 120 mm/s extrusion speed, 20% filling) (Figure 3).

### 2.3. Pre-Contoured Plate

**Figure 4:**
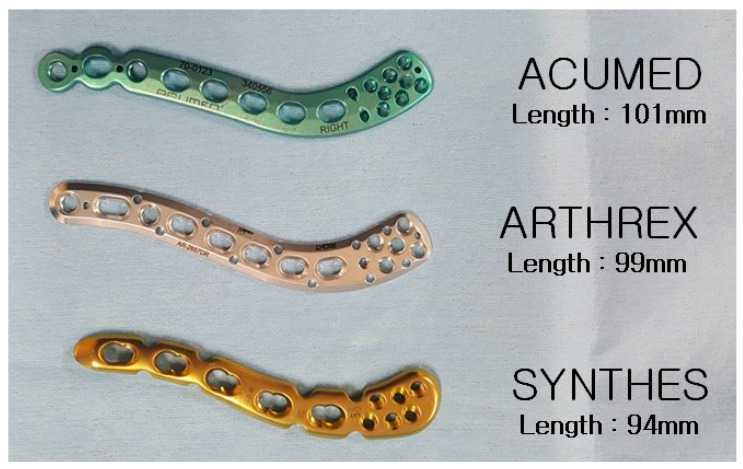
Three products of pre-contoured anatomical distal clavicle plate systems.

Among the pre-contoured plates available on the market, 3 products of anatomical distal clavicle plate systems (Acumed (Hillsboro, OR, USA), Synthes (West Chester, PA, USA), and Arthrex (Naples, FL, USA)) were applied to the model. A plate approximately 10 cm in length was selected for measurement from each company to standardize the comparison (Figure 4).

A hypothetical fracture line was created at the distal third of the clavicle. The choice of a 10 cm plate length was based on the need to ensure adequate fracture working space while providing fixation of 6 cortices distally. The specific plate used for this setup was a Synthes 5-hole plate, measuring 94 mm in length. This 94 mm plate was used as a reference and compared with plates from other companies that had similar lengths.

Each plate was pre-contoured in an S-shape along the long axis to follow the clavicle’s outline. Along the short axis, two plates (Acumed and Arthrex) were flat while one plate (Synthes) was pre-contoured.

### 2.4. Measurement Techniques

**Figure 5:**
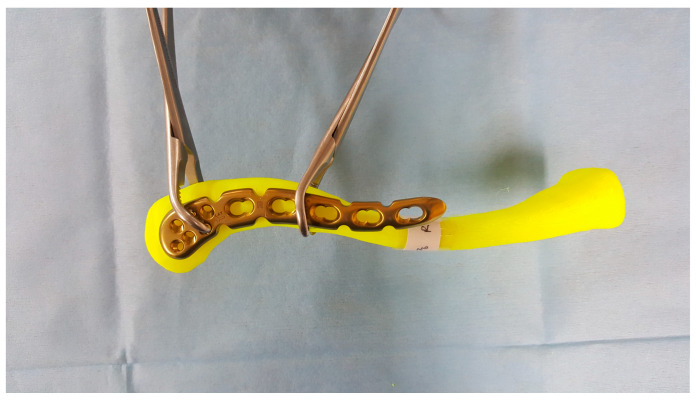
The pre-contoured plate was placed over the distal clavicle where it fit best and fixed with clamps.

**Figure 6:**
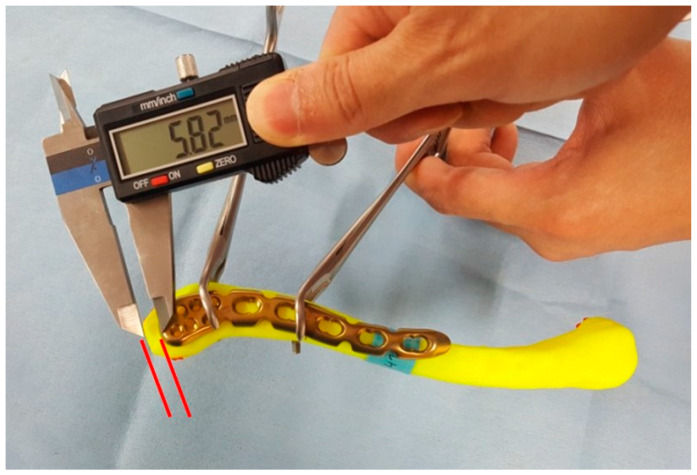
Lateral distance of clavicle distal edge to plate lateral end.

Thirty clavicle models were classified into 6 groups according to the medio-lateral length of the clavicle. Considering that the main purpose of the pre-contoured plate is proper fixation of the plate to the clavicle without additional manipulation, pre-contoured distal clavicle plates were mounted on the superior aspect of the clavicle model. To simulate an actual intraoperative situation, the plates were placed and adjusted to best fit the clavicle model and then secured with surgical clamps (Figure 5). The distal edge of the clavicle typically has a protruding shape, which complicates plate placement and is also meaningful in the aspect of preserving the acromioclavicular joint. To determine the ideal location for mounting the pre-contoured plate, the distance from the distal edge of the clavicle to the lateral end of the plate was measured using a digital caliper (Figure 6).

**Figure 7:**
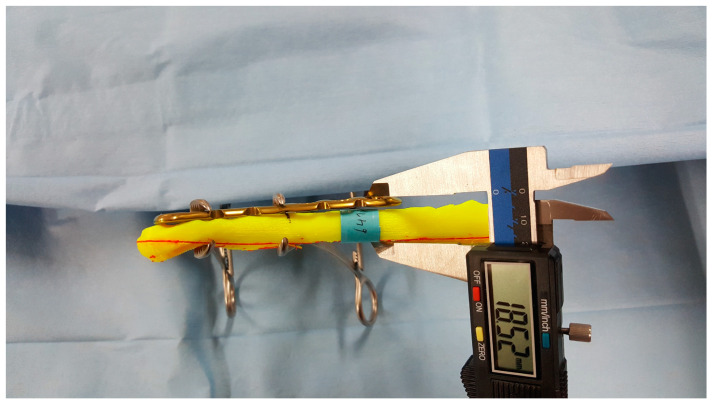
The medial gap between the clavicle and plate.

Skin protrusion caused by the plate can lead to pain, discomfort, and cosmetic issues. To account for this, the gap between the clavicle and the plate was analyzed by subtracting the diameter of the clavicle from the distance between the plate’s superior surface and the inferior border of the clavicle (Figure 7).

**Figure 8:**
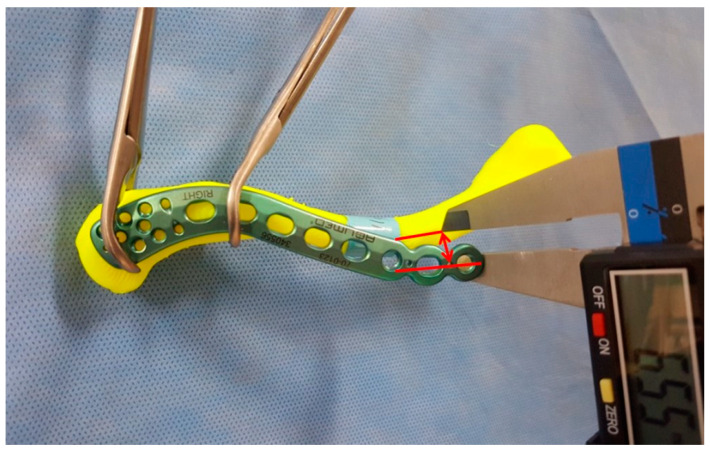
The overhang of the plate to the clavicle bone.

The overhang of the plate relative to the clavicle bone model was measured as the distance between the end point of protruding plate and the center of the clavicle in the superior view (Figure 8).

### 2.5. Statistical Analysis

Statistical analysis of data was conducted using IBM-SPSS Statistics version 24.0 (SPSS Inc., Chicago, IL, USA). The Kolmogorov–Smirnov test was used for normality testing. Descriptive analysis, Student’s *t*-test and paired *t*-test were used to analyze the age and length of the clavicle. Distance between the lateral edge of plate and the clavicle, the gap between the clavicle surface and the plate, and the overhang were compared between male and female and among 3 types of plate system with an ANOVA test. The Kruskall–Wallis test was used for subgroup analysis. The significance level for all analyses was set at *p* < 0.05.

## 3. Results

### 3.1. Patient Demographics

**Figure 9:**
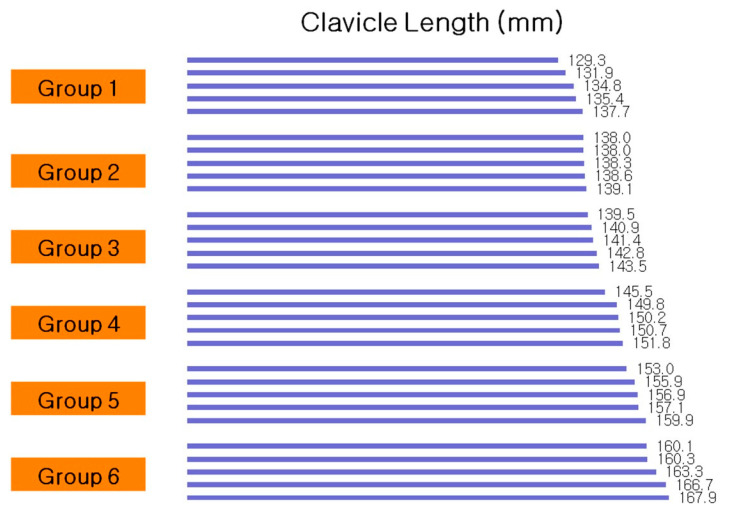
The 6 groups categorized according to the mediolateral length of the clavicle.

The mean age of male subjects was 50.5 ± 18.2 years and female subjects was 62.5 ± 17.7 years. The mean clavicle length in the male group was 156.1 ± 7.6 mm and in the female group, it was 138.4 ± 4.3 mm. Both groups showed normal distribution (*p* > 0.05 for each male and female), and there was a statistically significant difference in clavicle length between men and women (Table 1). To facilitate subgroup analysis without considering sex variables, the clavicle models were divided into six groups based on their mediolateral length. The shortest models were categorized into Group 1 (Figure 9).

### 3.2. Lateral Distance in the Coronal Plane between the Lateral End of Clavicle Model and Plate

The distance between the lateral end of the clavicle model and the plate was compared across different products of clavicle plate systems and between men and women (Table 2). The mean distance was 7.9 ± 1.7 mm, with a mean of 8.1 ± 1.8 mm for men and 7.7 ± 1.5 mm for women. The largest distance was 8.6 ± 2.2 mm (Synthes, men), and the smallest was 7.3 ± 1.2 mm (Acumed, women). There was no significant difference found between sexes and among the three products of pre-contoured plates (Table 2). When the models were divided into six groups based on the length of the clavicle, the distance between the lateral end of the clavicle model and the plate did not show significant differences between the products of the clavicle plate systems (Table 3). In terms of mounting the pre-contoured plate on the clavicle model where it best fits, no statistical difference was observed in the lateral distance in the coronal plane between sexes or among products.

### 3.3. The Medial Gap between the Clavicle and Plate

**Figure 10:**
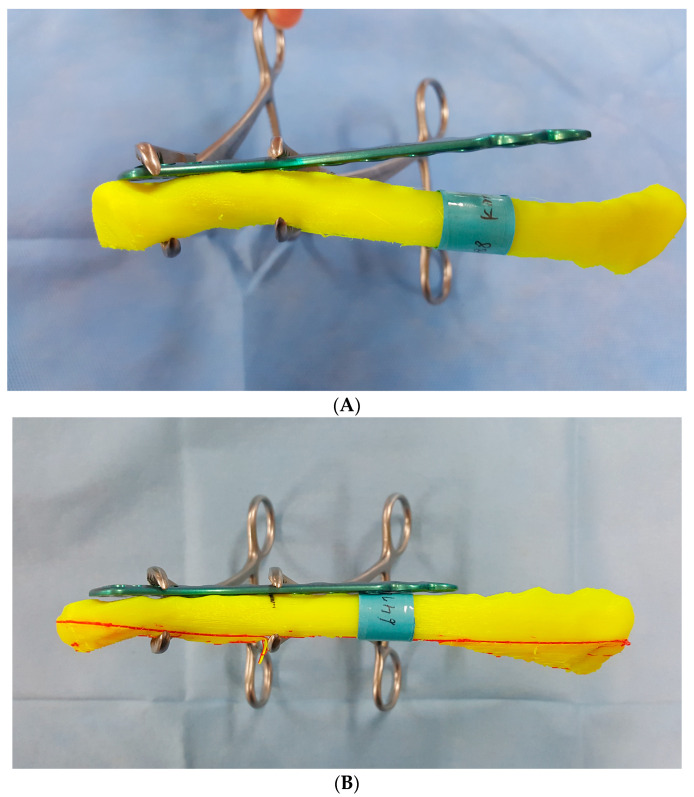
(**A**) Large medial gap with a pre-contoured plate temporarily fixed with surgical clamps on a shorter female clavicle model. (**B**) Minimal gap with a pre-contoured plate temporarily fixed with surgical clamps on a longer male clavicle model.

The mean distance was 3.6 ± 3.0 mm with a mean of 2.9 ± 2.4 mm for men and 4.3 ± 3.3 mm for women. The largest value observed was 4.6 ± 3.7 mm (Acumed, women), while the smallest was 4 ± 2.6 mm (Arthrex, men). No significant differences were found between men and women or among the three types of pre-contoured plates (Table 4). When the data were categorized into six groups based on the length of the clavicle, the largest gap was observed in Group 2 across all three products. The smallest gap was found in Group 3 for two products (Synthes and Acumed) and in Group 1 for one product (Arthrex). A statistically significant difference was found in one product (Acumed) among the groups. However, there was no significant difference between the three products (Table 5) (Figure 10).

### 3.4. The Medial Overhang of the Plate from Clavicle Bone Model

**Figure 11:**
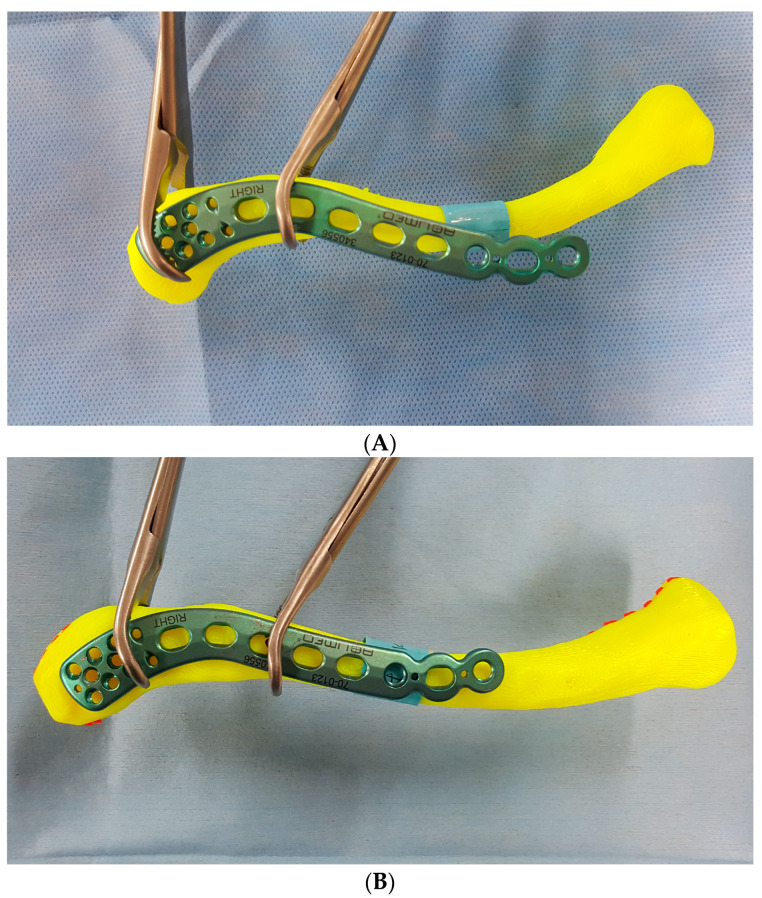
(**A**) Large overhang with a pre-contoured plate temporarily fixed with surgical clamps on a shorter female clavicle model. (**B**) Minimal overhang with a pre-contoured plate temporarily fixed with surgical clamps on a longer male clavicle model.

The mean distance was 5.8 ± 4.6 mm, with a mean of 3.9 ± 3.4 mm for men and 7.8 ± 4.8 mm for women. The largest value observed was 9.8 ± 4.5 mm (Arthrex, women), while the smallest was 3.4 ± 3.6 mm (Arthrex, men). Regarding the anterior or posterior overhang of the clavicle, no significant differences were found among the three products. However, within these products, statistically significant differences were found between men and women in Acumed (*p* = 0.037) and Arthrex (*p* = 0.000) plates. The overhang value was larger in both women’s groups (Table 6). When the data were categorized into six groups based on the clavicle length, the largest overhang value was found in Group 1, which represents the shortest clavicle length, across all products. Notably, the largest difference in overhang between products was observed in Group 3. Statistically significant differences were found in two products (Synthes and Arthrex) among the six groups. However, there was no significant difference among the three products overall (Table 7) (Figure 11).

## 4. Discussion

By using 3D-printed models, this study demonstrated significant individual differences in anatomic clavicle morphology. The pre-contoured clavicle plates showed low conformity and high variance among individuals. Additionally, the lateral distance between the distal end of the clavicle and the plate was not consistent, making it difficult to suggest an objectively optimal position. This study also identified the potential for a medial gap, which, if too large, could result in malreduction, necessitating additional bending. Furthermore, the possibility of overhang was observed, which could make it difficult to use the distal screw holes if the overhang is significant.

Superior clavicle plating, which is commonly chosen, carries risks such as underlying neurovascular injury, prominent skin protrusion, or hypertrophic scarring. To address these issues and accommodate the complex 3D anatomy of the human clavicle, a distal clavicle anatomical pre-contoured plate was developed. This plate aims to shorten operation time by eliminating the need for additional bending during surgery and reducing the risk of infection associated with plate repositioning. If the plate does not anatomically contour to the bone precisely, it can lead to nonunion or malunion of the fracture. Additionally, skin protrusion caused by an imperfectly contoured plate may result in discomfort and cosmetic problems, potentially requiring further surgery for plate removal.

Numerous studies have reported good clinical outcomes using pre-contoured plates. Fleming et al. [27] reported a 100% union rate and excellent return of function, with no mandatory need for removal. High union rates with good clinical outcomes and low complication rates were reported using superior locking pre-contoured plates in patients with distal clavicle fractures in a study by Andersen et al. [28]. Chandrasenan et al. [29] revealed that pre-contoured anatomical plates are fit to the actual clavicle and shorten the operation time. Additionally, their high mechanical strength decreases the likelihood of metal breakage. VanBeek et al. [30] reported that plates with low profiles and beveled edges help prevent skin protrusion, thereby reducing the necessity for plate removal.

However, despite these advantages, pre-contoured plates often do not fit the clavicle perfectly. This misfit is due to the complex anatomical features of the human clavicle and individual variations. A cadaveric study by Andermahr et al. [31] which analyzed the length, diameter, and curvature of 196 embalmed and 10 fresh human clavicles, revealed significant anatomical variations among individuals. When comparing sexes, male clavicles were found to be longer, thicker, and to have a greater curvature than female clavicles. Consistent with previous studies, the length of the clavicle differed significantly between men and women in this study.

In terms of the conformity between pre-contoured plates and human clavicle models, this study revealed significant discrepancies, particularly with larger gaps observed in shorter clavicles. These findings align with the study by Huang et al. [32]. which identified the apex of the superior bow on the lateral aspect of the clavicle as a challenging fit for pre-contoured plates. Furthermore, unlike in men, pre-contoured plates did not conform well in white women. Unlike our study, which analyzed Asian clavicle models, Huang et al.’s research focused on the pre-contoured Acumed Locking Clavicle, a design optimized for the medial three-fifths of the clavicular shaft. Their study employed a two-dimensional analysis using digital image software (Adobe Photoshop). Malhas et al. [8] suggested that smaller clavicles, commonly found in women, benefit more from plating systems with various plate shapes. Most anatomical studies have been conducted on Western populations, including white and black individuals, and the design of pre-contoured plates typically reflects these populations’ anatomical data. In contrast, our study used 3D models of Asian clavicles, which led to larger gaps and greater discrepancies in plate conformity.

Most anatomical studies on the human clavicle have been cadaveric or based on imaging software, such as Photoshop or 3D virtual reconstruction medical software using CT data [32,33]. Particularly, assessing the contact and compatibility of pre-contoured plates with the clavicle in simulation studies can be challenging, especially concerning screw fixation, because the contact situation may change after compression from screw fixation. This study is significant because it used actual clavicle models created with 3D printing technology instead of relying solely on image-based simulations. By using these actual models, it is possible to replicate the real conditions of plate fixation. Additionally, a bone clamp was used in this study to simulate the compression of the plate via screw fixation, providing a more accurate representation of the clinical scenario.

Over the past decade, 3D printing technology has been widely adopted in medicine. Particularly in orthopedics, it has been actively applied to implant design and the creation of surgical guides [34,35,36]. Prebending the pre-contoured clavicle plate prior to surgery and using the prebent plate during the operation can potentially shorten the operation time and is especially beneficial when employing the minimally invasive plate osteosynthesis (MIPO) technique. Kim et al. [3] demonstrated that preoperative prebending of pre-contoured plates, compared to intraoperative bending, resulted in better anatomical reduction and shorter surgery times in patients with mid-shaft clavicle fractures. Additionally, another study by Kim et al. [37] utilized 3D-printed clavicle models to improve the fitting of pre-contoured plates in comminuted fractures, which facilitated minimally invasive procedures and enhanced fracture healing.

The current study had multiple limitations. First, the plating and measurement were performed by a senior surgeon, whose subjectivity may have influenced the process. Although the surgeon has extensive experience with clavicle fractures, the surgical concept and perspective of a single individual could have impacted the objectivity of this study. However, efforts were made to ensure objectivity by using clamping to replicate the plate and clavicle mismatch encountered during actual surgeries. The experience of a seasoned senior surgeon was utilized to enhance the reliability of the measurements. Second, only the vertical and horizontal lengths between the clavicle model and the pre-contoured plate were measured and analyzed. Due to the use of 3D-printed models, three-dimensional measurements such as rotational or angular assessments were not conducted. Third, the Meshmixer software was used for smoothing to address step-off artifacts in the reconstructed model. However, differences or variations were not verified using specific features. The authors minimized the smoothing process to modify only the step-off artifacts and preserve the original structure. Fourth, only three products from the anatomical distal clavicle plate system were evaluated. Different results might be obtained with other products. Nevertheless, this study is significant because it focused on pre-contoured anatomical distal clavicle plate systems, in contrast to previous studies that analyzed plates suitable for the mid-shaft or medial three-fifths of the clavicle [29,30,32,33]. Fifth, the sample size was relatively small, with only 30 cases, which may limit the generalizability to the entire population. However, the clavicle lengths of both men and women in this study followed a normal distribution, with 100% post hoc power [38].

## 5. Conclusions

There were significant variations in clavicle length between sexes and among individuals in the 3D-printed clavicle models. Pre-contoured plates, which are designed to conform to clavicle anatomy, showed notable discrepancies, particularly in shorter clavicles.

## Figures and Tables

**Table 1:** Demographical data showed men had longer clavicles compared to women.

Sex	Age (Years)	Clavicle Length (mm)
Men (*n* = 15)	50.5 ± 18.2	156.1 ± 7.6
Women (*n* = 15)	62.5 ± 17.7	138.4 ± 4.3
*p*-value	0.079	0.000 *

* *t*-test.

**Table 2:** The lateral distance between the lateral end of the clavicle model and the plate (mm).

Sex	Acumed	Arthrex	Synthes	*p*-Value
Men	7.6 ± 1.6	8.2 ± 1.6	8.6 ± 2.2	0.315
Women	7.3 ± 1.2	7.5 ± 1.3	8.2 ± 1.7	0.185
*p*-value	0.590	0.212	0.331	

**Table 3:** The lateral distance between the lateral end of the clavicle model and the plate divided by the length (mm).

Group	Acumed	Arthrex	Synthes	*p*-Value
1	7.6 ± 1.1	7.0 ± 1.3	7.7 ± 2.0	0.675
2	7.7 ± 0.8	7.3 ± 1.1	9.1 ± 0.9	0.067
3	7.1 ± 1.7	7.6 ± 0.9	7.5 ± 1.0	0.120
4	6.2 ± 1.2	8.6 ± 1.3	8.7 ± 1.7	0.264
5	7.8 ± 1.1	7.9 ± 1.0	9.3 ± 2.2	0.393
6	8.3 ± 2.0	8.5 ± 2.5	8.3 ± 3.3	0.992
*p*-value	0.437	0.647	0.702	

**Table 4:** The medial gap between clavicle surface and plate (mm).

Sex	Acumed	Arthrex	Synthes	*p*-Value
Men	3.3 ± 2.5	2.4 ± 2.6	2.9 ± 2.2	0.653
Women	4.6 ± 3.7	4.0 ± 4.0	4.1 ± 2.1	0.866
*p*-value	0.244	0.216	0.118	

**Table 5:** The medial gap between the clavicle surface and plate divided by the length (mm).

Group	Acumed	Arthrex	Synthes	*p*-Value
1	2.0 ± 1.1	2.2 ± 2.5	4.2 ± 1.4	0.112
2	8.7 ± 2.5	8.3 ± 3.4	6.7 ± 1.4	0.230
3	3.4 ± 3.1	1.2 ± 1.0	2.1 ± 0.8	0.756
4	3.5 ± 2.4	2.7 ± 1.7	3.0 ± 1.5	0.827
5	2.3 ± 2.5	1.4 ± 1.1	2.4 ± 2.3	0.932
6	3.7 ± 2.7	3.5 ± 4.3	2.6 ± 2.1	0.612
*p*-value	0.043 ^†^	0.284	0.282	

^†^ Kruskall Wallis test.

**Table 6:** Overhang distance (mm/anterior or posterior).

Sex	Acumed	Arthrex	Synthes	*p*-Value
Men	4.6 ± 3.1	3.4 ± 3.6	3.5 ± 3.6	0.554
Women	7.8 ± 4.8	9.8 ± 4.5	5.9 ± 4.6	0.082
*p*-value	0.037 *	0.000 *	0.128	

* ANOVA test.

**Table 7:** Overhang distance (mm/anterior or posterior) divided by length (mm).

Group	Acumed	Arthrex	Synthes	*p*-Value
1	11.9 ± 5.0	13.6 ± 2.9	10.0 ± 4.1	0.330
2	5.9 ± 3.2	7.3 ± 4.0	5.0 ± 3.7	0.539
3	7.2 ± 2.4	10.3 ± 2.5	2.9 ± 3.0	0.077
4	3.3 ± 2.5	1.7 ± 1.0	1.1 ± 0.8	0.183
5	6.0 ± 3.5	3.7 ± 4.0	3.7 ± 3.4	0.307
6	3.2 ± 2.8	3.0 ± 3.6	5.4 ± 4.7	0.612
*p*-value	0.057	0.008 ^†^	0.034 ^†^	

^†^ Kruskall Wallis test.

## Data Availability

The raw data supporting the conclusions of this article will be made available by the authors on request.
